# Supplementary material for: High school science fair: School location trends in student participation and experience
Source: PLoS One. 2023 Sep 11;18(9):e0291049. doi: 10.1371/journal.pone.0291049 (PMC10495023; doi:10.1371/journal.pone.0291049)
Supplement: S1 Table — (PDF) [file pone.0291049.s003.pdf]

Supplemental Table 1. Student survey answers year-to-year

Survey Questions	Answers	Year (# Students*)		
		2020 (900)	2021 (583)	2022 (951)
		Student Answers %		
1. What grade are you in?	9th	43.2	45.3	41.1
	10th	34.0	31.0	32.9
	11th	16.9	17.7	17.9
	12th	5.8	5.8	7.9
2. Location of high school	Suburban	73.4	72.6	75.1
	Urban	21.6	25.7	22.0
	Rural	5.0	1.7	2.9
3. Gender?	Male	40.8	39.5	44.2
	Female	59.0	59.9	53.3
4. Ethnicity most identified with?	Asian	35.4	35.5	33.3
	Black	8.8	11.5	9.3
	Hispanic	17.2	19.2	15.0
	White	33.8	28.0	37.4
	Other	4.1	5.3	4.2
5. During high school have you carried out science fair more than once?	Yes	38.0	41.9	31.9
	No	61.9	58.1	67.6
6. In which science fair competitions did you compete this year?	School	50.1	40.7	46.9
	District	8.9	14.2	14.4
	Regional	22.0	27.3	21.3
	State	1.4	1.5	2.6
7. Was your science fair project Team or Individual?	Individual	58.8	74.4	63.4
	Team	37.8	21.8	33.8
8. Was the science fair project required by your school?	Yes	63.8	53.9	65.4
	No	19.0	26.9	16.4
	Satisfied School Project	14.3	15.6	15.4
9. Do you think science fair projects should be optional or required? (Need not be for competition.)	Optional	75.6	76.5	74.0
	Required	23.1	21.6	24.6
10. Do you think science fair projects for competition should be optional or required?	Optional	83.9	85.6	87.3
	Required	13.8	12.2	11.5
	1. Parents	48.0	45.5	49.2

11. Who actually helped you?	2. Siblings	10.9	12.2	12.7
	3. Other family members	5.0	4.3	4.7
	4. Teachers	52.4	49.7	52.7
	5. Other students	30.7	18.9	31.9
	6. Scientists	8.1	7.2	6.2
	7. A paid mentor	0.3	0.7	1.1
	8. Articles on the Internet	57.9	61.1	57.3
	Articles in books or magazines	22.9	28.1	20.4
	Other	3.0	3.9	3.0
12. What kind of help did you actually receive?	1. Being given the main idea	9.0	8.2	11.1
	2. Development of the idea	26.7	28.8	31.0
	3. Gathering background research information, or finding a research site or participants	25.1	23.0	44.3
	4. Performing the experiments	28.7	23.7	35.6
	5. Writing the report	9.1	8.2	13.1
	6. Fine tuning the report after it is written	33.2	35.5	29.0
	7. Designing the poster board and presentation	22.2	12.3	20.1
	8. Producing charts or graphs	14.1	10.5	16.3
	9. Coaching for the interview with judges	20.8	19.4	9.9
	10. Copying the project from someone else	0.7	0.3	0.7
	Other	6.1	8.7	4.8
13. Did you get the kind of help you wanted from teachers?	Yes	74.8	76.7	81.7
	No	24.2	21.4	15.4
14. Did you get the amount of help you wanted from teachers?	Yes	73.4	75.6	80.4
	No	25.0	22.0	16.5
15. What types of communications did you use in your science fair presentation?	Written report	0.0	0.0	50.8
	Literature review	0.0	0.0	15.4
	Research notebook	0.0	0.0	30.5
	Poster board presentation	0.0	0.0	59.0
	Power point presentation	0.0	0.0	44.2
	Software to show results	0.0	0.0	45.4
	Interview with judges	0.0	0.0	40.7
	Other	0.0	0.0	2.3
16. What obstacles did you face?	1. Coming up with the main idea	45.8	43.6	44.4
	2. Getting motivated to do the project	43.2	41.2	46.8
	3. Becoming disappointed with the project	26.1	20.2	22.9
	4. Limited resources	38.9	39.1	31.8
	5. Limited knowledge	31.6	29.7	24.5
	6. Limited skills	25.0	21.3	17.7
	7. Limited cooperation	12.9	9.4	11.6
	8. Getting organized	26.3	23.8	25.1

	9. Time pressure	63.0	60.4	58.9
	10. Not enough money	17.1	10.6	9.5
	11. Results not as expected	22.7	17.0	25.0
	Other	3.4	3.9	3.5
17. How did you overcome the obstacles?	1. Used someone else's main idea	0.9	1.7	1.3
	2. Picked a familiar / interesting topic	31.0	32.2	30.9
	3. Did more background research	50.1	54.0	44.8
	4. Stopped working on the project for a while	15.8	17.2	17.9
	5. Made a timeline to follow	22.7	29.3	25.9
	6. Perseverance and self-discipline	48.4	49.7	50.1
	7. Had someone else to keep me on track	15.9	15.1	14.4
	8. Had someone else do the math	1.4	1.2	0.3
	9. Changed the research plan	19.1	22.6	17.7
	10. Collected more data	24.2	27.3	24.7
	11. Had someone else collect the data	2.2	0.7	1.3
	12. Used someone else's data	0.6	1.5	0.6
	13. Made up the data	3.6	2.2	1.6
	14. Changed the hypothesis to fit the data	3.9	4.8	3.0
	15. Changed the data to fit the hypothesis	1.6	1.9	1.1
	Other	4.4	3.9	4.5
20. Are you interested in a career in the sciences or engineering?	Yes	55.3	60.9	58.4
	No	19.1	14.4	16.1
	Not Sure	25.4	24.5	25.6
21. Did your science fair experience increase your interest in the sciences or engineering?	Yes	55.9	57.8	51.9
	No	43.7	41.5	46.9
*Number of incomplete surveys not included: 2020, 138; 2021, 102; 2022, 189				

Year	2020	2021	2022
Surveys included	900	583	951
Surveys incomplete	88	67	152
Surveys no location data	50	36	37
Total excluded	138	103	189
