# Supplementary material for: High school science fair: School location trends in student participation and experience
Source: PLoS One. 2023 Sep 11;18(9):e0291049. doi: 10.1371/journal.pone.0291049 (PMC10495023; doi:10.1371/journal.pone.0291049)
Supplement: S2 Table — (PDF) [file pone.0291049.s004.pdf]

Supplemental Table 3. School location, ethnicity, and SEF experience

Survey Questions	Answers	School Location		p Value
		Suburban	Urban	
		Student Answers % (#)		
**Asian Students #**		**681**	**154**	
Who helped you with your SEF project? (more than one answer possible)	Parents	46.3 (315)	41.6 (64)	0.290
	Teachers	51.4 (350)	51.9 (80)	0.901
	Scientists	10.4 (71)	10.4 (16)	0.989
Kind of help received	Fine tuning report	29.5 (201)	36.4 (56)	0.096
	Coaching interview	21.6 (147)	16.9 (26)	0.193
Highest level of SEF competition?	School	42.8 (257)	56.6 (69)	0.038
	District	14.8 (89)	10.7 (13)	
	Regional	38.6 (232)	31.1 (38)	
	State	3.8 (23)	1.6 (2)	
SEF Increased Interest	Yes	68.4 (466)	61.0 (94)	0.078
Interest in S&E Career	Yes	70.6 (481)	60.4 (93)	0.041
	No	8.2 (56)	12.3 (19)	
	Not Sure	21.1 (144)	27.3 (42)	
**Black Students #**		**159**	**65**	
Who helped you with your SEF project? (more than one answer possible)	Parents	42.1 (67)	41.5 (27)	0.934
	Teachers	47.8 (76)	49.2 (32)	0.846
	Scientists	2.5 (4)	3.1 (2)	0.813
Kind of help received	Fine tuning report	28.9 (46)	27.7 (18)	0.852
	Coaching interview	10.7 (17)	13.8 (9)	0.504
Highest level of SEF competition?	School	69.7 (85)	73.2 (41)	0.831
	District	10.7 (13)	7.1 (4)	
	Regional	18.9 (23)	19.6 (11)	
	State	0.8 (1)	0.0 (0)	
SEF Increased Interest	Yes	39.6 (63)	41.5 (27)	0.791
Interest in S&E Career	Yes	49.7 (79)	43.1 (28)	0.632
	No	26.4 (42)	29.2 (19)	
	Not Sure	23.3 (37)	27.7 (18)	
**Hispanic Students #**		**225**	**158**	
Who helped you with your SEF project? (more than one answer possible)	Parents	42.7 (96)	38.6 (61)	0.426
	Teachers	51.1 (115)	49.4 (78)	0.737
	Scientists	6.7 (15)	6.3 (10)	0.895
Kind of help received	Fine tuning report	31.1 (70)	21.5 (34)	0.038
	Coaching interview	17.3 (39)	13.9 (22)	0.369
Highest level of SEF competition?	School	43.5 (84)	49.6 (65)	0.627
	District	23.3 (45)	22.9 (30)	

	Regional	30.6 (59)	24.4 (32)	
	State	2.6 (5)	3.1 (4)	
SEF Increased Interest	Yes	59.6 (134)	60.1 (95)	0.911
Interest in S&E Career	Yes	58.7 (132)	51.9 (82)	0.384
	No	18.2 (41)	19.6 (31)	
	Not Sure	23.1 (52)	28.5 (45)	
**White Students #**		**641**	**151**	
Who helped you with your SEF project? (more than one answer possible)	Parents	56.2 (360)	51.7 (78)	0.316
	Teachers	51.2 (328)	55.0 (83)	0.401
	Scientists	4.1 (26)	8.6 (13)	0.02
Kind of help received	Fine tuning report	36.2 (232)	41.7 (63)	0.206
	Coaching interview	12.9 (83)	15.9 (24)	0.341
Highest level of SEF competition?	School	67.0 (349)	64.6 (82)	0.709
	District	12.9 (67)	13.4 (17)	
	Regional	18.4 (96)	22.0 (28)	
	State	1.7 (9)	0.0 (0)	
SEF Increased Interest	Yes	42.1 (270)	42.4 (64)	0.953
Interest in S&E Career	Yes	52.7 (338)	49.0 (74)	0.512
	No	21.1 (135)	19.2 (29)	
	Not Sure	26.2 (168)	30.5 (46)	
