# Supplementary material for: High school science fair: School location trends in student participation and experience
Source: PLoS One. 2023 Sep 11;18(9):e0291049. doi: 10.1371/journal.pone.0291049 (PMC10495023; doi:10.1371/journal.pone.0291049)
Supplement: S3 Table — (PDF) [file pone.0291049.s005.pdf]

Supplemental Table 2. School location, gender, and SEF experience

Survey Questions	Answers	Location		p Value
		Suburban	Urban	
		Student Answers % (#)		
**Males #**		**777**	**203**	
Who helped you with your SEF project? (inclusive)	Parents	52.3 (406)	43.8 (89)	0.033
	Teachers	48.9 (380)	52.7 (107)	0.334
	Scientists	8.9 (69)	7.4 (15)	0.499
Highest level of SEF competition?	School	45.9 (357)	49.8 (101)	0.105
	District	13.9 (108)	10.3 (21)	
	Regional	24.6 (191)	20.2 (41)	
	State	2.6 (20)	0.5 (1)	
SEF increased your interest in S&E	Yes	57.8 (449)	57.1 (116)	0.869
Interest in an S&E Career	Yes	65.1 (506)	57.1 (116)	0.110
	No	12.9 (100)	15.8 (32)	
	Not Sure	22.0 (171)	27.1 (55)	
**Females #**		**997**	**344**	
Who helped you with your SEF project? (inclusive)	Parents	47.5 (474)	43.3 (149)	0.175
	Teachers	53.3 (531)	51.5 (177)	0.563
	Scientists	5.0 (50)	7.6 (26)	0.079
Highest level of SEF competition?	School	47.1 (470)	48.3 (166)	0.688
	District	11.0 (110)	13.1 (45)	
	Regional	23.1 (230)	20.9 (72)	
	State	1.7 (17)	1.7 (6)	
SEF increased your interest in S&E	Yes	52.2 (520)	52.0 (179)	0.969
Interest in an S&E Career	Yes	55.6 (554)	49.7 (171)	0.159
	No	18.8 (187)	20.1 (69)	
	Not Sure	25.6 (255)	29.9 (103)	
